# miR-214-Enriched Extracellular Vesicles Released by Acid-Adapted Melanoma Cells Promote Inflammatory Macrophage-Dependent Tumor Trans-Endothelial Migration

**DOI:** 10.3390/cancers14205090

**Published:** 2022-10-18

**Authors:** Elena Andreucci, Jessica Ruzzolini, Francesca Bianchini, Giampaolo Versienti, Alessio Biagioni, Matteo Lulli, Daniele Guasti, Patrizia Nardini, Simona Serratì, Francesca Margheri, Anna Laurenzana, Chiara Nediani, Silvia Peppicelli, Lido Calorini

**Affiliations:** 1 Department of Experimental and Clinical Biomedical Sciences “Mario Serio”, Università degli Studi di Firenze, 50134 Florence, Italy; 2 Department of Experimental and Clinical Medicine, Università degli Studi di Firenze, 50134 Florence, Italy; 3 Laboratory of Nanotecnology, IRCCS Istituto Tumori “Giovanni Paolo II”, 70124 Bari, Italy

**Keywords:** acidic tumor microenvironment, melanoma, extracellular vesicles, miR-214, inflammation, vascular permeability, trans-endothelial migration

## Abstract

**Simple Summary:**

Cutaneous melanoma is the most aggressive form of skin cancer with high-metastatic ability. Despite the recent advancements in melanoma treatments, the prognosis of metastatic patients remains very poor. A better understanding of the molecular mechanisms leading to melanoma dissemination is urgently needed in order to develop novel therapeutical strategies to ameliorate patients’ outcomes. Extracellular vesicles (EV) released by tumor cells are key players in metastasis development: by conveying bioactive molecules with oncogenic activity, they can modulate the surrounding—and even the distant—microenvironment and reprogram recipient cells to facilitate the metastatic cascade. Here, we show that melanoma cells release a higher amount of miR-214-enriched EV when adapted to extracellular acidosis, which promote a macrophage activation state, capable of facilitating the trans-endothelial migration of melanoma cells. Thus, we disclose a new molecular mechanism to prevent melanoma intravasation based on miR-214 targeting.

**Abstract:**

The understanding of the molecular mechanisms leading to melanoma dissemination is urgently needed in view of the identification of new targets and the development of innovative strategies to improve patients’ outcomes. Within the complexity of tumor intercellular communications leading to metastatic dissemination, extracellular vesicles (EV) released by tumor cells are central players. Indeed, the ability to travel through the circulatory system conveying oncogenic bioactive molecules even at distant sites makes EV capable of modulating recipient cells to facilitate metastatic dissemination. The dynamic remodeling of the tumor microenvironment might influence, along with a number of other events, tumoral EV release. We observed that, in melanoma, extracellular acidosis increases the release of EV enriched in miR-214, an onco-miRNA involved in melanoma metastasis. Then, miR-214-enriched EV were found to induce a state of macrophage activation, leading to an overproduction of proinflammatory cytokines and nitric oxide. Such an inflammatory microenvironment was able to alter the endothelial cell permeability, thereby facilitating the trans-endothelial migration of melanoma cells, a crucial step in the metastatic cascade. The use of synthetic miR-214 inhibitors and miR-214 overexpression allowed us to demonstrate the key role of miR-214 in the EV-dependent induction of macrophage activation. Overall, our in vitro study reveals that the release of tumor miR-214-enriched EV, potentiated by adapting tumor cells to extracellular acidosis, drives a macrophage-dependent trans-endothelial migration of melanoma cells. This finding points to miR-214 as a potential new therapeutic target to prevent melanoma intravasation.

## 1. Introduction

Cutaneous melanoma is the most lethal form of skin cancer, accounting for more than 320,000 cases and about 57,000 cancer-related deaths per year worldwide [1]. The incidence of melanoma is globally increasing more rapidly than any other kind of cancer [2]. If not removed early by surgical resection, cutaneous melanoma disseminates very rapidly leading to metastatic disease and poor patient outcomes. The 5-year survival rate for patients affected by stage IV melanoma is dramatically nearby 15%, and despite the recent introduction of new therapeutic options, such as immuno- and targeted therapy, which has significantly decreased the mortality rate, much is still needed to make melanoma curable [3].

Extracellular vesicles (EV) are crucial mediators within the complex array of cell–cell interactions involved in the several steps of the metastatic cascade. EV are nano-sized membranous structures released by most cells into the extracellular space, including body fluids [4]. Based on their size, EV are classified as exosomes (diameter 30–100 nm), microvesicles (also called ectosomes; 100–1000 nm), and apoptotic bodies (1000–5000 nm) [5]. EV are involved in several biological functions, such as the removal of harmful cellular materials, trophic support, and cell–cell communication. Indeed, the ability of EV to travel through body fluids—conveying functional information to distant sites in vivo—and infiltrate the biological barriers make them capable to drive intercellular communication in all tissues in both physiologic and pathologic conditions [6]. This not only changed completely the concept of intercellular communication but also clarified several cellular processes in cancer [7]. EV cargos comprise classical soluble and insoluble signaling factors, such as structural proteins, lipids, and nucleic acids, including microRNAs (miRNAs).

miRNAs are small single-stranded RNAs, with an average of 22 nucleotides in length, belonging to the family of non-coding RNAs. miRNAs affect DNA, RNA, and proteins acting as negative regulators of gene expression, being able to control the translation and even the transcription of target mRNAs. Actually, miRNAs act as molecular sponges, influencing multiple biological processes, including cell proliferation, differentiation, migration, angiogenesis, and apoptosis, and their dysregulated expression is associated with a plethora of diseases, including cancer [8]. All of this makes miRNAs an attractive target for oncology research [9].

Among the melano-miRNAs (i.e., miRNAs involved in melanoma formation and progression), miR-214 has been found to promote melanoma metastasis by increasing migration, invasion, extravasation, and survival of melanoma cells into the circulatory system [10,11,12]. Notably, miR-214 targeting has been recently proposed as a promising anti-metastatic therapy [13].

Here, we show that miR-214, conveyed by EV particularly released when melanoma cells were adapted to extracellular acidosis, exerts a proinflammatory activity able to alter the vascular structures to facilitate the trans-endothelial migration of melanoma cells. Extracellular acidosis is now recognized as a crucial aspect of the tumor microenvironment (TME), which is central in cancer progression, including melanoma. These findings provided mechanistic insight into the role of miR-214 in melanoma progression, strengthening the possibility to exploit miR-214 targeting to prevent or treat metastatic disease.

## 2. Materials and Methods

### 2.1. Cell Culture and Treatments

B16-F10 (hereafter referred to as B16) were kindly supplied by Dr S. Gattoni-Celli (Medical University of South Carolina, Charleston, SC, USA) [14]. B16-LU and B16-LI murine melanoma cell lines were previously isolated in our laboratory from B16-derived lung and liver metastasis, respectively, in a syngeneic metastatic experimental model. Briefly, B16-LU and B16-LI were obtained by the so-called experimental metastasis assay consisting of the intravenous injection of B16 murine melanoma cells into C57Bl/6 syngeneic mice. The primary A375 and Sk-Mel-2 cell lines, the metastatic WM-266-4 human melanoma cell line, and the murine macrophage RAW 264.7 cell line were purchased by American Type Culture Collection (ATCC; Rockville, MD, USA). The murine endothelial cells were kindly given by Dr. Ilaria Cimmino and Professor Francesco Oriente (Department of Translational Medicine, Research Unit (URT) Genomic of Diabetes, Institute of Experimental Endocrinology and Oncology, National Council of Research (CNR), University of Naples Federico II, Naples, Italy). All the cell lines used were grown at a 37 °C, 5% CO_2_ humidified atmosphere in DMEM 4.5 g/L glucose, 2 mM L-glutamine, and 10% FBS (Euroclone, Pero, Italy) (decomplemented FBS was used for culturing RAW 264.7 macrophages). Extracellular acidosis was mimicked in vitro by culturing melanoma cells in pH 6.7 ± 0.1 medium for at least 3 months before use, as previously described [15]. MISSION^®^ Synthetic microRNA Inhibitors anti-miR-214-3p and anti-miR-214-5p were purchased by Merck Life Science S.r.l. and administered to RAW 264.7 macrophages in the presence of EV to interfere with miR-214 activity. miR-214 Mouse MicroRNA Expression Plasmid and the empty vector were purchased by OriGene Technologies GmbH (Herford, Germany) and transfected through Lipofectamine™ 3000 Transfection Reagent (Thermo Fisher Scientific, Milan, Italy) in RAW 264.7 cells. The selection of miR-214-overexpressing cells (miR-214+) was performed by geneticin treatment (Merk Life Science S.r.l., Milan, Italy) followed by cell sorting for GFP with a BD FACS Melody (BD Biosciences, Milan, Italy) flow cytometer. Lipopolysaccharide (LPS, 10 ng/mL) was administered to RAW 264.7 macrophages as a positive control of the proinflammatory macrophage activation. 1 × 10^6^ RAW 264.7 cells were seeded into 6-well plates; treated or not for 16 h with EV ± anti-miR-214 or LPS; and conditioned media (CM) was collected for either IL-1β, IL-6, and TNF-α ELISA or nitric oxide (NO) detection (in this case, DMEM 4.5 g/L glucose without phenol red was used) or for a 24-h treatment of murine endothelial cells.

### 2.2. Extracellular Vesicles (EV)

#### 2.2.1. Isolation of EV through the Ultracentrifugation Method

Melanoma cells were starved from FBS for 24 h, and 30 mL of the culture medium was collected and subjected firstly to 300× *g* for 5 min to discard the cell pellet and then to 3000× *g* for 30 min at 4 °C to remove cell debris and apoptotic bodies. The supernatant was then subjected to 10,000× *g* at 4 °C for 1 h and 100,000× *g* at 4 °C for 1 h to isolate the ectosome and exosome fractions, respectively. Based on their final destination, the ectosome and exosome fractions were resuspended in PBS or culture medium and mixed in a single EV-containing solution.

#### 2.2.2. Transmission Electron Microscopy

Samples were prepared for transmission electron microscopy (TEM) by the negative and positive staining procedures. In brief, the EV mixtures comprising exosomes and ectosomes were fixed in Karnovsky’s fluid for 5 min at room temperature (RT), centrifuged for 5 min at 11,000× *g*, and then rinsed and resuspended in Cacodylate buffer 0.2 M. Aliquots of these suspensions were sedimented for 5 min at RT or 15 min at 37 °C on 300 mesh, and copper/formvar-coated grids depending on the kind of staining (respectively, negative and positive). The Uranyless (Electron Microscopy Sciences, Hatfield, PA, USA) was used both as negative and positive staining for 5 RT and 15 min at 37 °C. Dried samples were analyzed at a Jem 1010 transmission electron microscope (Jeol, Tokyo, Japan) at 80 Kv and photomicrographs acquired with a MegaView III digital camera (Soft Imaging System, Muenster, Germany) and analyzed with AnalySIS software (Soft Imaging System, Muenster, Germany).

#### 2.2.3. Nanoparticle Tracking Analysis

A nanoparticle tracking analysis (NTA) was performed, as previously described [16], with a NanoSight NS300 (Malvern Panalytical, Westborough, MA, USA) apparatus equipped with a 488-nm excitation laser and an automated syringe sampler. According to the NanoSight technology, the EV size is calculated through the Stokes–Einstein equation based on the relationship between the Brownian motion and hydrodynamic diameter. The EV samples were diluted at 1:500 in PBS and loaded into 1-mL syringes. CSV files generated by NTA by software v3.2 were used for a computational analysis.

### 2.3. RNA Isolation and Quantitative Real-Time Polymerase Chain Reaction (qPCR)

#### 2.3.1. miRNA

Total RNA was prepared using Tri Reagent (Merk Life Sciences S.r.l., Milan, Italy), agarose gel checked for integrity, and quantified at the NanoDrop™ 8000 Spectrophotometer (Thermo Fisher Scientific, Milan, Italy). Reverse transcription was performed with the MystiCq^®^ microRNA cDNA Synthesis Mix (Merck Life Science S.r.l., Milan, Italy), and miR-214 expression was evaluated at the Bio-Rad CFX96 Touch™ Real-Time PCR Detection System (Bio-Rad, Milan, Italy) using MystiCq^®^ microRNA^®^ SYBR^®^ Green qPCR ReadyMix™ (Merck Life Science S.r.l., Milan, Italy) according to the manufacturer’s instructions. MystiCq^®^ miR-214-3p/5p and qPCR Assay Primers and MystiCq^®^ microRNA qPCR Control Primer (RNU-6) were purchased from Merck Life Science S.r.l.

#### 2.3.2. mRNA

Total RNA was prepared using Tri Reagent (Merk Life Sciences S.r.l., Milan, Italy), agarose gel checked for integrity, and quantified with the NanoDrop™ 8000 Spectrophotometer (Thermo Fisher Scientific, Milan, Italy). Reverse transcription was performed with the iScript cDNA Synthesis Kit (Bio-Rad, Milan, Italy) according to the manufacturer’s instructions. Selected genes were evaluated at the Bio-Rad CFX96 Touch™ Real-Time PCR Detection System (Bio-Rad, Milan, Italy) using SsoAdvanced Universal SYBR Green Supermix (Bio-Rad, Milan, Italy). The primer sequences (Merck Life Science S.r.l., Milan, Italy) are the following: IL-1β FW: CCT GCA GCT GGA GAG TGT GGA; IL-1β RV: CCC ATC AGA GGC AAG GAG GAA; IL-6 FW: CTT CCA TCC AGT TGC CTT CT; IL-6 RV: TGC ATC ATC GTT GTT CAT AC; TNF-α FW: GCG GTG CCT ATG TCT CAG CC; TNF-α RV: TGA GGA GCA CGT AGT CGG GG; 18S FW: CGC CGC TAG AGG TGA AAT TCT; 18S RV: CGA ACC TCC GAC TTT CGT TCT; β-ACTIN FW: CAT TGC TGA CAG GAT GCA GAA GG; β-ACTIN RV: TGC TGG AAG GTG GAC AGT GAG G.

### 2.4. Immunofluorescence

RAW 264.7 cells were seeded on glass coverslips in six-well plates and treated with EV isolated from B16, B16-LU, and B16-LI cell lines under control pH or chronic acidic conditions. RAW 264.7 macrophages were fixed for 30 min at 4 °C with 3.7% paraformaldehyde and permeabilized for 15 min with PBS 0.1% Triton X-100 at RT. After 1-h incubation in blocking buffer (0.1% Triton X-100 and 5.5% horse serum PBS), cells were stained with anti-NF-kB p65 (GeneTex, purchased from Prodotti Gianni, Milan, Italy) for 1 h at RT and then 45 min at RT in the dark with Cy3-conjugated anti-mouse antibody (Thermo Fisher Scientific, Milan, Italy). Following the 20-min nuclei staining with DAPI (Thermo Fisher Scientific, Milan, Italy) at RT in the dark, cells were mounted onto glass slides and visualized at the SP8 confocal microscope (Leica Microsystems, Milan, Italy). Manders coefficients by ImageJ software, proportional to the amount of fluorescence of the colocalizing pixels in NF-ĸB and DAPI color channels, were used to calculate the NF-ĸB nuclear fraction.

### 2.5. Western Blot

Melanoma-derived EV and RAW 264.7 macrophages were lysed in radioimmunoprecipitation assay (RIPA) lysis buffer (Merck Millipore, Milan, Italy) added with Pierce Protease Inhibitor Tablets (Thermo Fisher Scientific, Milan, Italy) for protein isolation. The protein concentration was measured with Bradford reagent (Merck Millipore, Milan, Italy), and equal amounts of protein were separated in Laemmli buffer on 8%–12% (*v/v*) SDS-PAGE gel (Thermo Fischer Scientific, Milan, Italy) and transferred to a polyvinylidene difluoride (PVDF) membrane using the iBlot 2 System (Thermo Fischer Scientific, Milan, Italy). Following 5-min incubation with EveryBlot Blocking Buffer (Bio-Rad, Milan, Italy), the membranes were probed overnight at 4 °C with anti-CD81 (sc-166029, Santa Cruz Biotechnology, Santa Cruz, CA, USA), anti-COX-2 (#4842S, Cell Signaling Technology, Danvers, MA, USA), and anti-tubulin (#3873, Cell Signaling Technology, Danvers, MA, USA) antibodies. Membranes were then incubated for 1-h RT with goat anti-mouse IgG Alexa Fluor 680 antibody or goat anti-rabbit IgG Alexa Flour 750 antibody (Thermo Fisher Scientific, Milan, Italy) and visualized at the Odyssey Infrared Imaging System (LI-COR^®^ Bioscience, Lincoln, NE, USA).

### 2.6. Enzyme-Linked Immunosorbent Assay (ELISA)

The IL-1β, IL-6, and TNF-α concentrations were measured in 100 μL of culture medium from RAW 264.7 cells treated with EV (with or without anti-miR-214) or 10 ng/mL LPS (CM) by mouse uncoated ELISA kits (Thermo Fisher Scientific, Milan, Italy) according to manufacturer’s instructions. The absorbance was measured at 450 nm at a microplate reader (BioTek, Winooski, VT, USA). The amount of each cytokine in the media was interpolated within the standard curves (GraphPad Prism 7 software). The results were normalized to the number of cells.

### 2.7. Nitric Oxide (NO) Assay

A Griess reaction was used to measure the NO concentration in the culture medium of RAW 264.7 macrophages. Briefly, 100 μL of the culture medium conditioned by RAW 264.7 cells treated with EV (with or without anti-miR-214) or 10 ng/mL LPS was mixed 1:1 with the Griess reagent (1% sulfanilamide, 0.1% N-1-naphthalenediamine dihydrochloride, and 2.5% H_3_PO_4_, Merk Life Sciences S.r.l., Milan, Italy) and transferred to 96-well plates. After 10 min of incubation at RT, the absorbance was measured at 540 nm with a microplate reader (BioTek, Winooski, VT, USA). A sodium nitrite (NaNO_2_) standard curve was used to calculate the amount of nitrite in each sample. The results were normalized to the number of cells.

### 2.8. Lactate Measure

The Lactate Colorimetric Assay Kit (BioVision, purchased from Vinci-Biochem, Florence, Italy) was used according to the manufacturer’s instructions to measure lactate production in the conditioned media (CM) of RAW 264.7 macrophages following 24-h treatment with EV. Data normalization was obtained by directly counting the number of cells to get a final result of lactate production (nM) by 1 × 10^5^ cells.

### 2.9. Seahorse Analysis

A number of (5 × 10^4^ RAW 264.7) cells treated for 24 h with EV were seeded onto Seahorse XFe96 microplates and evaluated with the Seahorse XFe96 Extracellular Flux Analyzer (Seahorse Bioscience, Billerica, MA, USA) for their glycolytic metabolism using the Glycolytic Rate Assay Kit (Agilent Technologies, Santa Clara, CA, USA), according to the manufacturer’s instructions. All the experiments were performed at 37 °C and normalized via cell protein measure with a Pierce BCA Protein Assay Kit (Thermo Fisher Scientific, Milan, Italy). The Seahorse XF Report Generator automatically calculated the parameters from wave data that were exported to GraphPad Prism software.

### 2.10. Flow Cytometry

Cells were harvested by Accutase (Euroclone, Pero, Italy), collected in flow cytometer tubes (2 × 10^5^ cells/tube), and stained for 1 h at 4 °C with anti-VE-cadherin F-8 conjugated with Alexa Fluor 488 (sc-9989 AF488; Santa Cruz Biotechnology, Santa Cruz, CA, USA). Following a wash in PBS, the cells were analyzed at BD FACSCanto II (BD Biosciences, Milan, Italy), calibrated by using cells incubated with Alexa Fluor 488-conjugated irrelevant IgG, and 1 × 10^4^ events/sample were analyzed.

### 2.11. In Vitro Permeability Assay

Millicell Cell Culture Inserts (Merck Life Sciences S.r.l., Milan, Italy) were placed onto 24-weel plates and polycarbonate filters coated with 0.25 µg/µL Matrigel. Some (1 × 10^5^) murine endothelial cells were cultured to confluency and treated for 24 h with the CM of RAW 264.7 previously subjected to EV treatment. Then, the permeability treatment was removed and albumin–fluorescein isothiocyanate conjugate (BSA-FITC, Merk Life Sciences, Milan, Italy) was incubated for 60 min. Some (100 μL) of the medium was collected from the receiver tray, transferred to a 96-well plate, and read on the Fluoroskan Ascent FL fluorescent plate reader (ThermoFiher Scientific, Milan, Italy) at 485 nm (excitation) and 535 nm (emission).

### 2.12. In Vitro Trans-Endothelial Migration

The trans-endothelial migration ability of B16 melanoma cells was evaluated towards murine endothelial cells monolayers pretreated for 24 h with CM of RAW 264.7 macrophages, previously receiving melanoma-derived EV. Briefly, Millicell Cell Culture Inserts (Merck Life Sciences S.r.l., Milan, Italy) were placed onto 24-weel plates, and the polycarbonate filters with 8 µm-diameter pores were coated with 0.25 µg/µL Matrigel. Some (1.0 × 10^5^) endothelial cells were cultured to confluency in the upper compartment. The day before the experiment, B16 cells were labeled with carboxyfluorescein diacetate succinimidyl ester (CFDA-SE, Thermo Fisher Scientific, Milan, Italy). Some (2.5 × 10^4^) CFDA-SE labeled B16 cells were seeded into the upper chamber and allowed to migrate for 6 h without any FBS gradient. Migrated cells were fixed in methanol for 1 h at 4 °C and observed at the SP8 confocal microscope (Leica Microsystems, Milan, Italy).

### 2.13. Statistical Analysis

All data were obtained based on at least three independent experiments. Statistical analysis was performed with GraphPad Prism 6 software by *t*-test, one-way analysis of variance (ANOVA), and two-way ANOVA, as specified in each figure legend. Values are presented as mean ± standard deviation (SD). The *p*-values are presented as * *p* < 0.05, ** *p* < 0.01, and *** *p* < 0.001. Values are presented as the mean of independent experiments ± SD.

## 3. Results

### 3.1. Acid-Adapted Melanoma Cells Released a Higher Amount of miR-214-Carrying EV Compared to Controls

**Figure 1:**
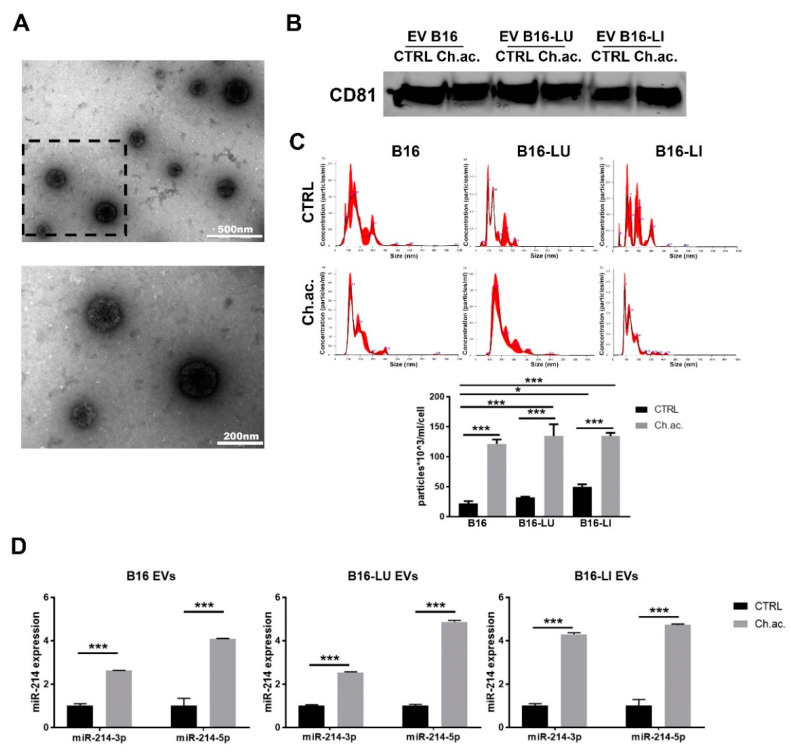
Extracellular vesicles released by melanoma cells. (**A**) Transmission electron microscopy (TEM) of B16-derived EV at different magnifications (upper picture: scale bar = 500 nm; lower picture: scale bar = 200 nm, magnification of the squared area above). (**B**) Western blot analysis of CD81 of melanoma-derived EV. (**C**) Nanoparticle tracking analysis (NTA) at Nanosight NS300 of EV released by B16, B16-LU, and B16-LI cells under standard (CTRL) and chronic extracellular acidosis (Ch.ac.) (one-way ANOVA). (**D**) miR-214-3p and miR-214-5p expression levels in EV released by B16, B16-LU, and B16-LI under standard and chronic acidic conditions (two-way ANOVA). * *p* < 0.05 and *** *p* < 0.001.

EV derived from B16, B16-LU, and B16-LI melanoma cells were evaluated by TEM (Figure 1A), evidencing a mixture of exosomes and microvesicles ranging from 50 to 400 nm in diameter, characterized by the expression of CD81 (Figure 1B; whole western blot membrane in Figure S1), one of the proper surface markers of EV. NTA revealed EV mixtures comprising exosomes of ≤100 nm in diameter and microvesicles of around 100–400 nm in length (Figure 1C). The mean values of the EV mixture released by each cell line under standard and chronic extracellular acidosis are 185.8 +/− 3.4 nm for control B16-derived EV vs. 174.4 +/− 2.5 nm for acid B16-derived EV, 150.9 +/− 14.3 nm for control B16-LU-derived EV vs. 208.6 +/− 7.9 nm for acid B16-LU-derived EV, and 185.5 +/− 10.1 nm for control B16-LI-derived EV vs. 142.9 +/− 3.2 nm for acid B16-LI-derived EV. Notably, for all the three cell lines, NTA highlighted a significant increase in EV release under extracellular acidosis compared to standard pH conditions, accounting for a ~five-fold increment in B16 cells, a ~four-fold increment in B16-LU cells, and a ~three-fold increment in B16-LI cells. Under standard pH conditions, no significant differences in the number of released EV were observed between the B16 and B16-LU cells, while B16-LI produced about a double amount of EV compared to B16 cells. Concerning acid EV, no significant differences were observed in the concentration of EV released by the three cell lines (Figure 1C, lower). Extracellular acidosis, besides affecting the amount of EV produced by each cell line, was found to also influence their cargos. In particular, the level of miR-214, a key “melano-miR” involved in melanoma progression and, conveyed in acid EV, was significantly higher than in control EV: miR-214-3p was increased ~2.5-fold in B16 and B16-LU acid-EV and ~4-fold in B16-LI acid-EV compared to control; miR-214-5p, despite less represented than the 3p strand, was upregulated in acid-EV of ~four-fold in all B16, B16-LU, and B16-LI-derived EV (Figure 1D). The increased miR-214 level in acid-EV was also observed in the A375, WM266-4, and Sk-Mel-2 human melanoma cell lines (Supplementary Figure S2).

### 3.2. miR-214-Enriched EV Released by Acid-Adapted Melanoma Cells Induce a Pro-Inflammatory Response in RAW 264.7 Macrophages

**Figure 2:**
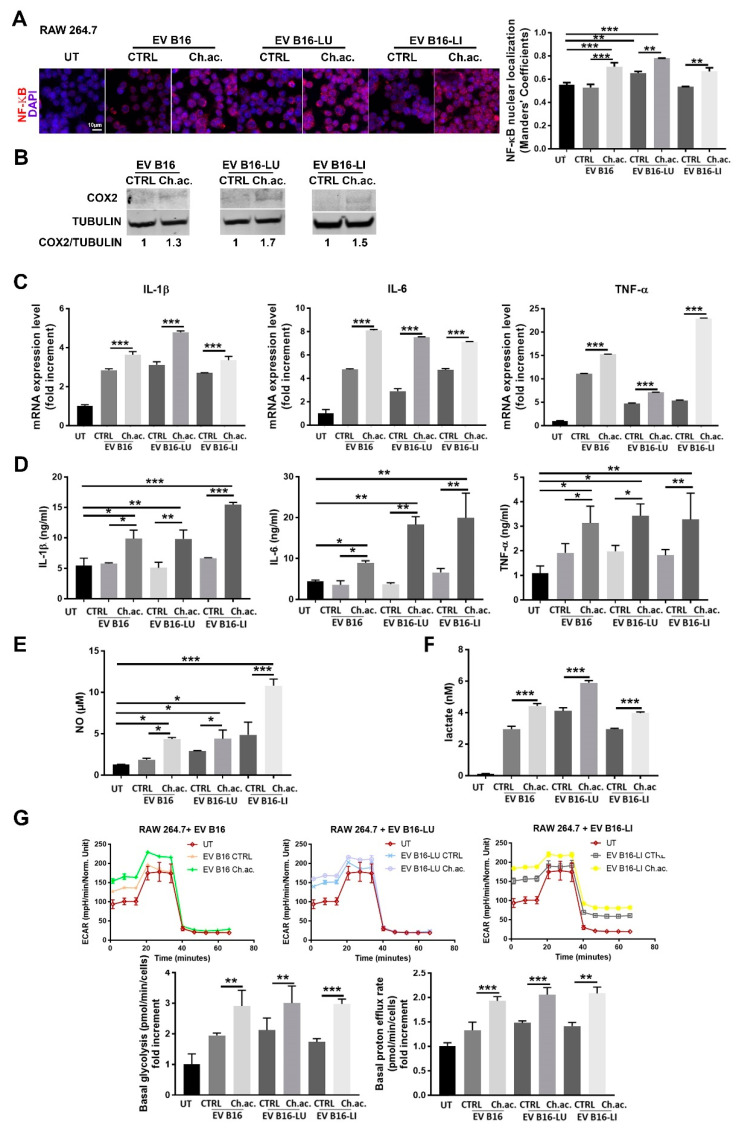
Inflammatory response of RAW 264.7 macrophages following the uptake of melanoma-derived acid-EV. (**A**) Immunofluorescence analysis of NF-ĸB nuclear localization in RAW 264.7 macrophages treated with melanoma-derived EV (Scale bar = 10 µm). (**B**) Western blot analysis of COX-2 in RAW 264.7 macrophages treated with melanoma-derived EV (*p* < 0.05). (**C**) Real-time qPCR of IL-1β, IL-6, and TNF-α in RAW 264.7 macrophages treated with melanoma-derived EV (all conditions were found significantly different from the untreated (UT). (**D**) ELISA of IL-1β, IL-6, and TNF-α released by RAW 264.7 macrophages treated with melanoma-derived EV. (**E**) NO production by RAW 264.7 macrophages treated with melanoma-derived EV. (**F**) Lactate concentration in CM of RAW 264.7 macrophages treated with melanoma-derived EV; all conditions were found significantly different compared to the untreated (UT). (**G**) Glycolytic Rate Assay as determined by Seahorse XFe96 analysis, with representative ECAR plots (**upper**), basal glycolysis (lower, **left**), and proton efflux rate (lower, **right**); all conditions were found significantly different from the untreated (UT). (one-way ANOVA; * *p* < 0.05, ** *p* < 0.01, and *** *p* < 0.001).

There is increasing evidence that EV play a critical role in reprogramming not only tumor cells but also innate and adaptive immune cells of TME, among which macrophages are particularly abundant and present at all stages of tumor progression, playing either tumor suppression or tumor potentiation roles. We found that the uptake of melanoma-derived acid EV by RAW 264.7 macrophages induced their activation towards a pro-inflammatory phenotype. The nuclear translocation of the NF-ĸB transcription factor was observed in RAW 264.7 macrophages upon the uptake of EV derived from acid-adapted B16, B16-LU, and B16-LI cells. This translocation, on the contrary, was not visible when treated with control EV. Nuclear NF-ĸB staining was indeed ~1.4-fold higher in macrophages treated with acid EV compared to the ones treated with those released by melanoma cells under standard pH conditions and to the untreated RAW 264.7 macrophages. A slight but significant increase in nuclear NF-ĸB localization was also observed following the uptake of control B16-LU-derived EV compared to the untreated macrophages (Figure 2A). Moreover, we observed a moderately increased expression of COX-2 in RAW 264.7 macrophages treated with acid melanoma-derived EV compared to those treated with control EV (Figure 2B; whole western blot membranes in Figure S1). The mRNA expression level of inflammatory cytokines such as IL-1β, IL-6, and TNF-α confirmed the macrophage activation state upon the treatment with the acid EV derived from all the three melanoma cell lines used. IL-1β and IL-6 mRNA were upregulated ~1.4-fold and ~2-fold, respectively, in RAW 264.7 macrophages treated with acid EV compared to control EV from B16, B16-LU, and B16-LI cells; TNF-α mRNA expression level was ~1.4-fold higher in macrophages treated with acid-EV than in those treated with control-EV from B16 and B16-LU cells, reaching a ~4-fold increase with acid EV produced by B16-LI melanoma cells. Compared to untreated RAW 264.7 macrophages, even the treatment with control EV from B16, B16-LU, and B16-LI cells stimulates the expression of IL-1β, IL-6, and TNF-α, at least at the mRNA level (Figure 2C). The production of these cytokines was then evaluated by ELISA, revealing that the treatment with control EV did not change IL-1β, IL-6, and TNF-α secretion by RAW 264.7 macrophages compared to their untreated condition but confirming a significantly increased production when subjected to the treatment with acid EV derived from the three melanoma cell lines. In other words, EV from acid-adapted B16 cells induce a higher production of IL-1β, IL-6, and TNF-α compared to EV derived from B16 cells grown at the control pH levels. Likewise, the acid EV derived from the two metastatic cell lines B16-LU and B16-LI enhanced the production of these cytokines compared to the control EV (Figure 2D). In parallel with the enhanced cytokine production, RAW 264.7 macrophages also produced an increased amount of NO when treated with acid EV released by B16 (~2.5-fold), B16-LU (~1.5-fold), and B16-LI (~2-fold) cells compared to control EV. The treatment with EV derived from B16 and B16-LU cells grown at standard pH did not alter NO production compared to the untreated RAW 264.7 macrophages, while those released by standard pH B16-LI cells promote a ~four-fold-increased production (Figure 2E). Macrophages adjust their cellular phenotype upon their activation also reprogramming their metabolism. Indeed, it is known that macrophage activation is accompanied by metabolic reprogramming towards glycolysis [17]. Following the uptake of acid EV produced by acid-adapted B16, B16-LU, and B16-LI cells, macrophages showed a boosted glycolytic metabolism when compared to macrophages treated with control EV, as witnessed by the doubled lactate production (untreated macrophages showed the lowest lactate production among all the conditions assessed, nearby the value of 0.2 nM, normalized on the number of producing cells) (Figure 2F). A deeper metabolic analysis performed with the Seahorse XFe96 analyzer using the Glycolytic Rate Assay kit revealed increased basal glycolysis coupled with a higher proton efflux rate in RAW 264.7 macrophages treated with acid EV compared to those receiving control EV (Figure 2G). These data confirmed that acid EV-induced macrophage activation is paralleled by a metabolic switch toward glycolysis. This last observation contributes to the characterization of the activated phenotype of acid EV-treated macrophages, in turn likely suggesting a tool to maintain a pro-tumoral extracellular acidosis. LPS treatment was used as a positive control of macrophage activation: RAW 264.7 cells treated with LPS showed a doubled increase in NF-ĸB nuclear staining (Supplementary Figure S3A), a dramatic overproduction of NO (Supplementary Figure S3B), and a boosted glycolysis as lactate production increases eight times compared to control/untreated cells (Supplementary Figure S3C) going in parallel with augmented basal glycolysis and an increased proton efflux rate (Supplementary Figure S3D).

### 3.3. miR-214 Interfering Counteracts the Pro-Inflammatory Response of Macrophages upon Acid-EV Uptake

**Figure 3:**
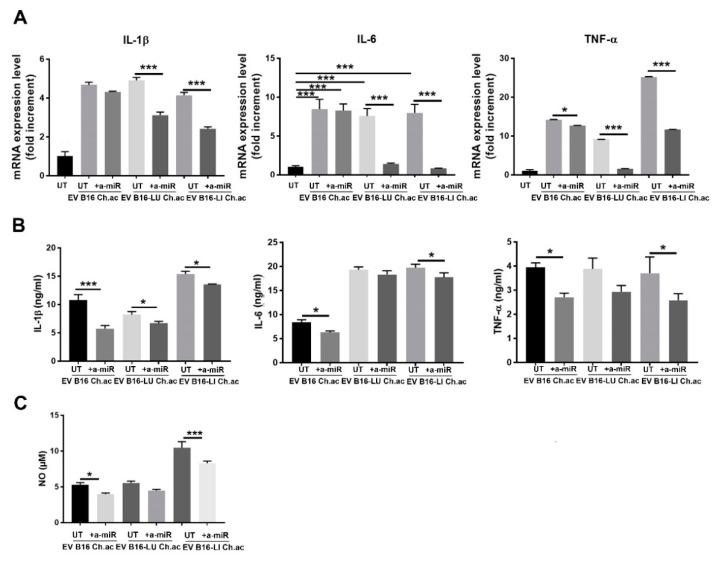
Anti-miR-214 treatment reverted the inflammatory phenotype induced in RAW 264.7 by melanoma-derived acid EV. (**A**) Real-time qPCR of IL-1β, IL-6, and TNF-α mRNA expression in RAW.264.7 treated with acid-EV derived from B16, B16-LU, or B16-LI cells in the presence or absence of anti-miR-214-3p/5p (a-miR). (**B**) ELISA for IL-1β, IL-6, and TNF-α production by RAW 264.7 treated with acid-EV in the presence or absence of anti-miR-214-3p/5p. (**A**,**B**) All conditions were found different (*p* < 0.005; one-way ANOVA) from the untreated (UT). (**C**) NO production by RAW 264.7 macrophages treated with acid EV in the presence or absence of anti-miR-214-3p/5p (one-way ANOVA; * *p* < 0.05, and *** *p* < 0.001).

By using anti-miRNA technology to inhibit miR-214 activities, we observed a reversion of the inflammatory state acquired by macrophages following the treatment with EV released by acid-adapted melanoma cells conveying high levels of miR-214. We firstly observed a diminished mRNA expression of IL-1β, IL-6, and TNF-α. In detail, the anti-miR-214, given together with acid EV from B16 cells, induced a slight decrease in IL-1β and IL-6 (even though not significant) and a ~2.5-fold decrease in TNF-α expression; when administered with B16-LU-derived and B16-LI-derived acid EV, the anti-miR-214 significantly decreased IL-1β, IL-6, and TNF-α (Figure 3A). By ELISA, we confirmed the impairment of the release of these cytokines, in particular, RAW 264.7 macrophages treated with B16-derived acid EV and anti-miR-214 reduced the production of IL-1β, IL-6, and TNF-α; following the treatment with acid EV derived from B16-LU cells in the presence of anti-miR-214, RAW 264.7 macrophages underwent a decreased production of IL-1β while IL-6 appeared unaffected; TNF-α production was instead reduced, although not significantly; RAW 264.7 macrophages treated with acid EV from B16-LI cells in the presence of anti-miR-214 showed a mild but significant decrease in the secretion of IL-1β and IL-6 and a ~1.5 fold--reduced production of TNF-α (Figure 3B). In line with these observations, NO production was significantly impaired by anti-miR-214 when administered together with acid EV from B16 or B16-LI cells, while no significance was obtained with acid EV from B16-LU cells, although a trend to decrease was visible (Figure 3C).

### 3.4. miR-214 Forced Expression in Macrophages Promotes the Pro-Inflammatory Phenotype

**Figure 4:**
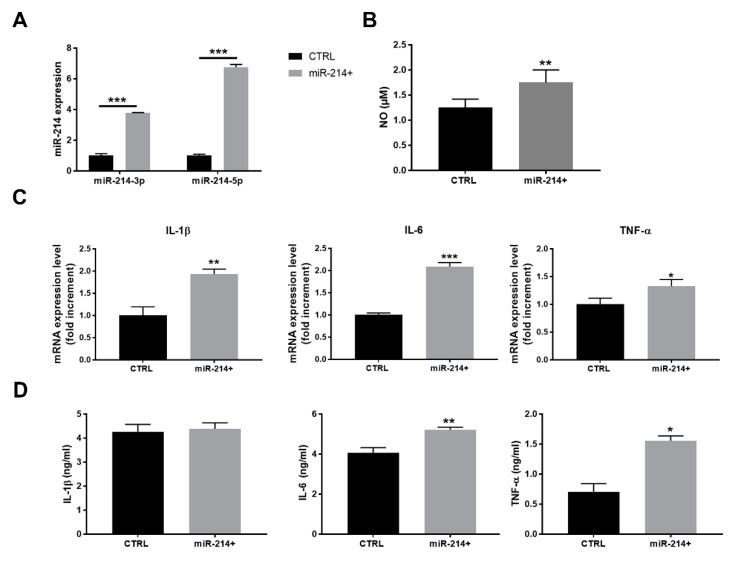
miR-214 over-expression induced the proinflammatory activation of RAW 264.7 macrophages. (**A**) Real-time qPCR of miR-214-3p/5p in RAW 264.7 macrophages where miR-214 was over-expressed compared to control cells (two-way ANOVA). (**B**) NO concentration in CM of control or miR-214-overexpressing RAW 264.7 macrophages (*t*-test). (**C**) Real-time qPCR of IL-1β, IL-6, and TNF-α mRNA in the control and miR-214-overexpressing RAW 264.7 macrophages (*t*-test). (**D**) ELISA of IL-1β, IL-6, and TNF-α released in CM of the control or miR-214-overexpressing RAW 264.7 macrophages (*t*-test). * *p* < 0.05, ** *p* < 0.01, and *** *p* < 0.001.

To strengthen the results obtained by anti-miRNA treatment, we adopted an opposite strategy determining whether a forced miR-214 expression in RAW 264.7 macrophages correlates with higher levels of proinflammatory activation. Once obtained miR-214-over-expressing (miR-214+) cells by genetic selection, we enriched the high-expressing population by cell sorting for GFP positivity. The quality of the procedure was verified by the real-time qPCR assessment of miR-214-3p/5p expression in the control and miR-214+ RAW 264.7 macrophages, showing a four-fold and a six-fold increase in 3p and 5p strand, respectively (Figure 4A). miR-214+ cells produced a higher amount of NO compared to the controls (Figure 4B), also displaying a ~2-fold increase in IL-1β and IL-6 mRNA expression and a ~1.4-fold increase in the TNF-α mRNA levels (Figure 4C). ELISA confirmed the overproduction of IL-6 and TNF-α in miR-214+ macrophages compared to control, while no significant variation was observed in IL-1β release (Figure 4D).

### 3.5. Conditioned Media of Acid-EV-Treated Macrophages Increase Vascular Permeability and Facilitate the Transendothelial Migration of Melanoma Cells

**Figure 5:**
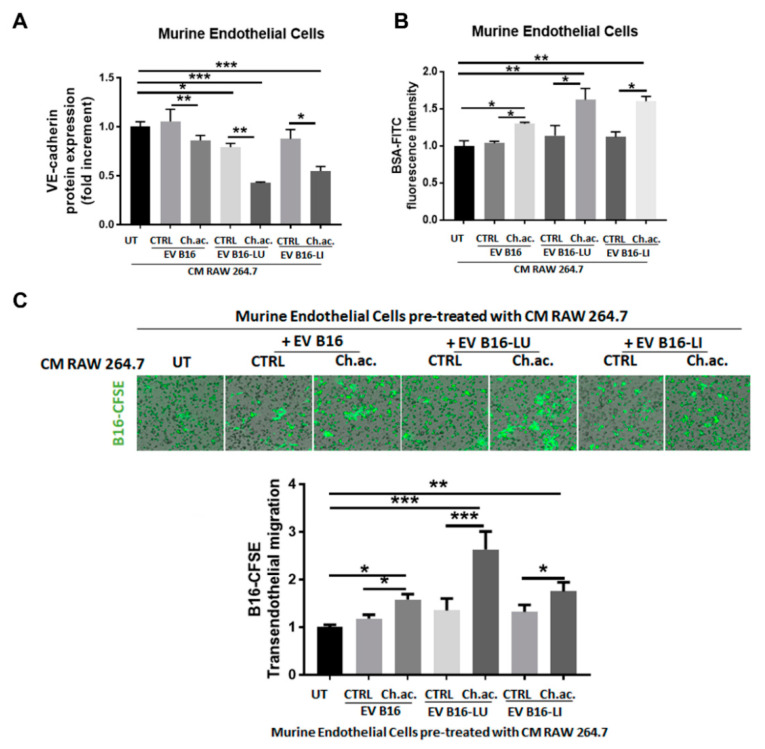
Vascular remodeling induced by the conditioned media of EV-treated macrophages. (**A**) Flow cytometric analysis of VE-cadherin protein expression in murine endothelial cells exposed to the CM of macrophages treated or not with melanoma-derived acid and control EV. (**B**) Permeability assay of murine endothelial cells exposed to the CM of macrophages treated or not with melanoma-derived acid and control EV. (**C**) Trans-endothelial migration of B16-CFSE melanoma cells through a monolayer of murine endothelial cells exposed to the CM of macrophages treated or not with melanoma-derived acid and control EV (one-way ANOVA; * *p* < 0.05, ** *p* < 0.01, and *** *p* < 0.001).

Inflammation is a strong inducer of vascular permeability, so we questioned whether the macrophage activation induced by melanoma-derived acid EV enriched in miR-214 can promote tumor progression by altering the integrity of the endothelial cell layer. This would facilitate the trans-endothelial migration of melanoma cells, increasing the tumor intravasation rate. To verify that, we let RAW 264.7 macrophages treated with melanoma-derived control and acid EV condition their culture media for 24 h, and then, we administered the collected CM to murine endothelial cells to evaluate any variation in vascular permeability. Endothelial cells receiving CM of macrophages treated with melanoma-derived acid EV, in contrast to control EV, showed an impaired plasma membrane expression of VE-cadherin, the most important adhesion molecule for the stability of endothelial intercellular junctions [18] (Figure 5A). This observation was further strengthened by the permeability assay data obtained showing that a significantly increased amount of BSA-FITC passes through the endothelial cell monolayer incubated with CM of RAW 264.7 macrophages treated with acid EV from B16, B16-LU, or B16-LI cells compared to the control EV (Figure 5B). Notably, such an increased vascular permeability facilitated B16 melanoma cell trans-endothelial migration (Figure 5C). These data suggest that the acid-adapted melanoma cells via the miR-214-enriched-EV release and subsequent macrophage activation orchestrate the establishment of an inflamed microenvironment that, in turn, disrupts the vascular integrity facilitating tumor cell intravasation, the first key step of the metastatic dissemination.

## 4. Discussion

The high proliferative rate of cancer cells is preferentially sustained by a glycolytic metabolism even in the presence of sufficient oxygen to guarantee phosphorylative oxidation, the so-called “Warburg effect” [19]. Such a boosted glycolysis leads to an excessive release of lactic acid in the extracellular milieu, inevitably causing the acidification of TME), further strengthened by the reduced lymphatic circulation and the high interstitial pressure typical of cancer tissues [20]. Thereby, almost all solid tumors experience an acidic extracellular pH ranging from 6.4 to 7.0 [21]. Huge evidence highlights that melanoma progression and metastatic dissemination are strongly promoted by the acidic TME. In the last decade, we and other research groups contributed to defining some of the tumor aggressive features fostered by extracellular acidosis, suggesting that the acidic TME affects each step of the metastatic cascade [22,23,24,25].

TME acidosis is known to participate actively in tumor progression and metastatic disease. Huge evidence highlights indeed that extracellular acidosis represents a perfect storm for metastasis development and it is now considered a new hallmark of cancer [25]. We previously reported that extracellular acidosis induces the acquisition of an extremely aggressive stem-like tumor phenotype endowed with a high ability to invade surrounding tissues, resist therapies, induce tumor vascularization, survive in the blood and lymphatic circulation, evade immune surveillance, and colonize secondary organs [15,23,24,26,27]. Notably, we showed that acid-adapted melanoma cells are less able to colonize secondary organs when injected alone into the mice’s tail vein but are capable to boost secondary organ colonization by the non-acid tumor cells when the co-injection of the two tumor sub-population was performed [28]. Such a contribution may likely rely on paracrine signals released by acid-adapted melanoma cells and received by the non-acid counterpart. In this context, EV may exert a crucial role. EV are emerging as critical messengers in cancer progression and metastasis [29]. By circulating through both blood and lymphatic vessels, EV create new hospitable sites for tumor growth, modulating inflammation, stromal organization, angiogenesis, coagulation, immune response, vascular permeability, and organotropism [7].

We observed that the release of EV by melanoma cells is strongly induced under extracellular acidosis, being acid-adapted melanoma cells capable to secrete a significantly higher number of EV compared to those cultured under standard pH conditions. This data is in line with previous findings showing that extracellular acidosis not only enhances the release of exosomes by tumor cells but also promotes significant modifications in the lipid component of the same particles [30,31,32]. Our data show that acid EV, compared to control EV, carry a high amount of miR-214, an onco-miRNA involved in the progression of different tumor types [13,33,34,35]. Focusing on melanoma, previous findings reported that miR-214 promotes the resistance to anoikis and the extravasation phases of the metastatic cascade [10,11,12]. In this study, we reveal a new possible way—promoted by the acidic TME—by which miR-214 can foster tumor trans-endothelial migration, acting not directly on melanoma cells but affecting macrophages. Macrophages are considered the principal regulators of several diseases, including cancer, and their accumulation in the tumor microenvironment is considered one of the hallmarks of cancer, also due to their plasticity to either reduce or potentiate tumor growth generating different local effectors. Indeed, we observed that miR-214 carried in EV released by acid-adapted melanoma cells is capable to induce a proinflammatory response in macrophages: upon NF-ĸB nuclear translocation together, macrophages increased the expression of COX-2 and the release of a high amount of IL-1β, IL-6, TNF-α, and NO, establishing an inflammatory microenvironment that in turn enhances vascular permeability facilitating melanoma cell trans-endothelial migration. In addition to NF-ĸB, COX-2, IL-1β, IL-6, TNF-α, and NO considered as markers of the activation phenotype expressed by macrophages exposed to miR-214-enriched EV, we also evaluated their metabolism. Generally, proinflammatory macrophages mainly rely on glycolysis and exhibit impaired tricarboxylic acid cycle and mitochondrial phosphorylative oxidation [36]. In our experimental model, we indeed observed that the proinflammatory macrophage activation induced by miR-214-enriched acid EV is coupled with a boosted glycolytic metabolism. The switch toward a glycolytic metabolism characterizing activated macrophages also offers the possibility to suggest a potential loop in which extracellular acidosis promotes the release of EV able to reprogram macrophage phenotype toward a prominent glycolytic activity sustaining, in turn, the acidosis of extracellular milieu.

The use of anti-miRNA oligonucleotides, on one hand, and of miR-214-overexpression, on the other, to interfere with or induce, respectively, miR-214-related changes allowed us to verify that miR-214 is a key mediator of the inflammatory macrophage response stimulated by acid-adapted melanoma cells through EV. Despite the roles of miR-214 in the regulation of inflammation being much less studied, there is evidence suggesting its involvement in inflammatory responses. Indeed, miR-214 was found upregulated in THP-1 cells and human monocytes treated with proinflammatory advanced glycation end products (AGEs) [37] and in activated T cells, playing a critical role in T-cell proliferation and excitation through targeting phosphatase and tension homolog deleted on chromosome 10 (PTEN) [38], known to have remarkable anti-inflammatory activities [39,40]. miR-214 overexpression was also found to induce an inflammatory response in the ulcerative colitis experimental model by promoting NF-kB phosphorylation and subsequently IL6 expression through PTEN and PDZ-LIM domain-containing protein 2 (PDLIM2) inhibition [41]. More recently, Zhao Li and colleagues disclosed the existence of a mutual suppression feedback loop between miR-214 and adenosine A_2A_ receptor (A_2A_R) signaling in the inflammatory response: briefly, they found that miR-214 overexpression promotes the release of inflammatory cytokines and, by directly binding to the 3′-UTR, downregulates A_2A_R expression; conversely, A_2A_R activity represses miR-214 expression in a PKA-NF-κB signaling-dependent manner. They also observed that NF-κB directly binds to the miR-214 promoter, resulting in transcriptional upregulation of miR-214 [42]. An in silico analysis by TargetScanHuman also suggests that, among putative miR-214 targets, besides PTEN, there are negative regulators of NF-ĸB, such as the NFKB inhibitor interacting Ras-like 2 (NKIRAS2) [43] and the NF-κB-repressing factor (NKRF), that specifically counteracts several basal NF-κB activities [44]. Thus, miR-214 represents a potential novel regulator of inflammation, despite the fact that its mechanisms of action in inflammation are not completely elucidated.

## 5. Conclusions

Overall, we revealed that the acidic TME potentiates the release by melanoma cells of miR-214-enriched EV that, in turn, promote the establishment of a macrophage-dependent inflammatory microenvironment facilitating vascular permeability and tumor trans-endothelial migration. Aware of the importance of translating this study in vivo, we plan to validate our results by using a model of experimental metastasis in syngeneic mice. In line with our observations, very recently, Bychkov and colleagues demonstrated that extracellular acidosis, via inducing metastatic melanoma cells to release EV with advanced pro-tumoral activity, sustained the acquisition of an increased malignant phenotype of melanoma cells themselves, also redirecting keratinocytes towards pro-tumoral activities [45]. This preclinical in vitro study allowed us to identify another mechanism through which the acidic TME may foster tumor progression and dissemination, exploiting the great potential of EV to convey pro-tumoral molecular signals capable of altering the microenvironment of secondary locations and prepare for metastatic colonization.

These data further strengthen the awareness of the pro-tumoral activity of the acidic TME, suggesting that miR-214-enriched EV potentiate melanoma intravasation.

## Data Availability

The data presented in this study are available in this article and supplementary materials.

## Supplementary Materials

The following supporting information can be downloaded at https://www.mdpi.com/article/10.3390/cancers14205090/s1: Figure S1: Western blot whole membranes. Figure S2: miR-214 in EV released by human melanoma cells. Figure S3: LPS-induced macrophage activation.
