# Supplementary material for: miR-214-Enriched Extracellular Vesicles Released by Acid-Adapted Melanoma Cells Promote Inflammatory Macrophage-Dependent Tumor Trans-Endothelial Migration
Source: Cancers (Basel). 2022 Oct 18;14(20):5090. doi: 10.3390/cancers14205090 (PMC9599952; doi:10.3390/cancers14205090)
Supplement: Supplementary file 1 [file cancers-14-05090-s001.zip › cancers-1882449-supplementary.pdf]

Supplementary Materials

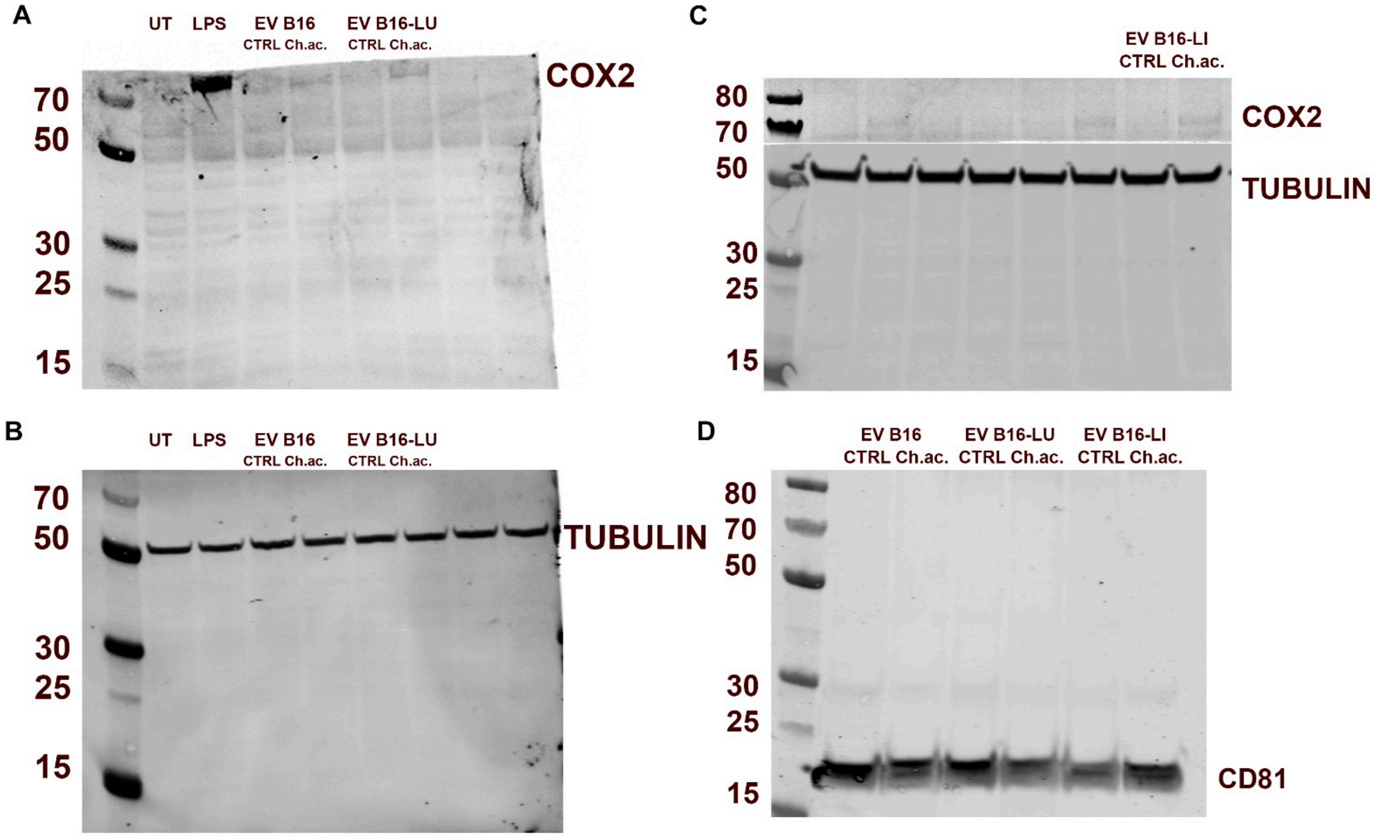

Figure S1. Western blot whole membranes. Images of whole western blot membranes of COX-2 (**A**) and relative tubulin (**B**) expression in RAW 264.7 macrophages treated with LPS or EV derived from control and acid B16 and B16-LU melanoma cells. (**C**) Western blot mebrane of COX-2 and tubulin expression in RAW 264.7 macrophages treated with EV derived from control and acid-adapted B16-LI melanoma cells. (**D**) Western blot membrane of CD81 in EV derived from control and acid-adapted B16, B16-LU and B16-LI melanoma cells.

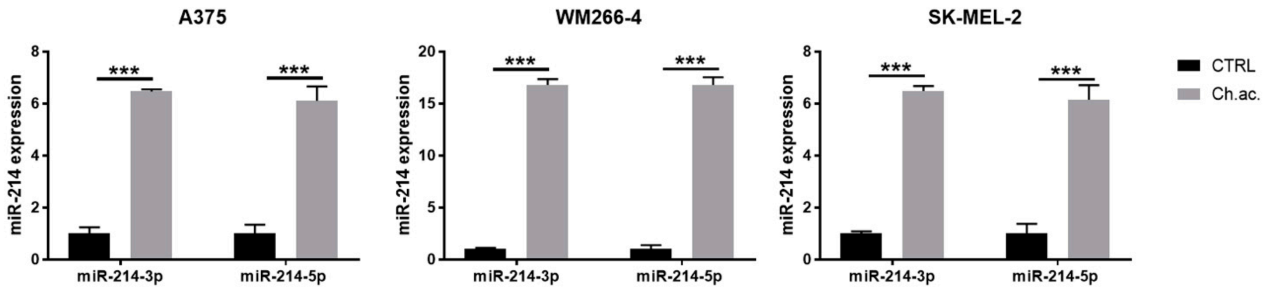

Figure S2. miR-214 in EVs released by human melanoma cells. Real-time qPCR analysis of miR-214-3p/5p levels carried in A375, WM266-4 and Sk-Mel-2 human melanoma cells grown under standard pH conditions (CTRL) or extracellular acidosis (Ch.ac.) (Two-way ANOVA).

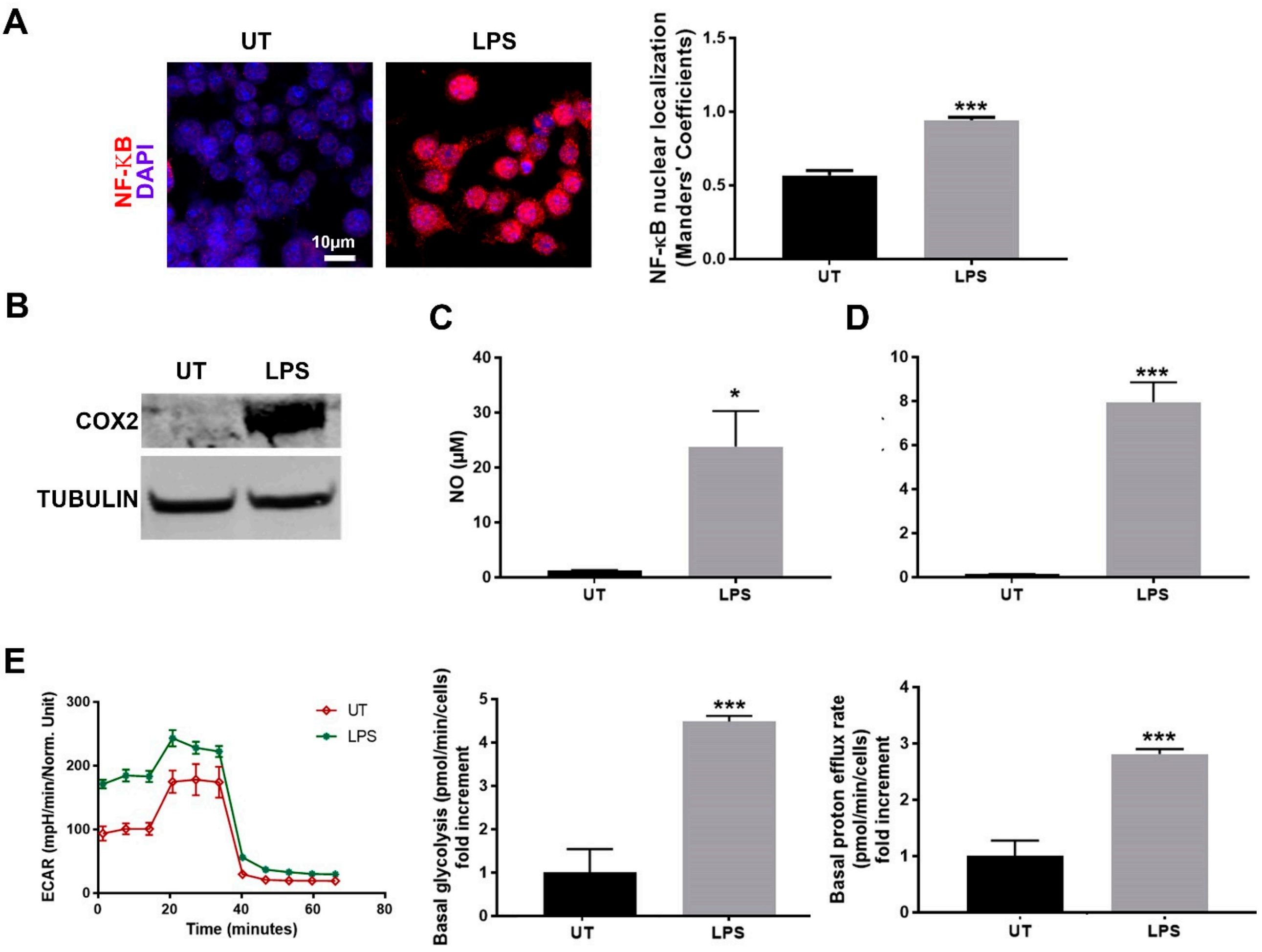

Figure S3. LPS-induced macrophage activation. (**A**) Immunofluorescence analysis of NF-κB nuclear localization in RAW 264.7 macrophages following LPS treatment (Scale bar = 10 µm; t-test). (**B**) Western blot analysis of COX-2 expression in RAW 264.7 treated with LPS. Tubulin served as housekeeper. (**C**) NO production by RAW 264.7 macrophages treated with LPS (t-test). (**D**) Lactate measure in conditioned media of RAW 264.7 treated with LPS (t-test) (**E**) Glycolytic Rate Assay at the Seahorse XFe96 analyzer, with representative ECAR plots (left), basal glycolysis (middle) and proton efflux rate (right) (t-test).
